# Managing Neovascular Age-Related Macular Degeneration in Clinical Practice: Systematic Review, Meta-Analysis, and Meta-Regression

**DOI:** 10.3390/jcm11020325

**Published:** 2022-01-10

**Authors:** Daniele Veritti, Valentina Sarao, Valentina Soppelsa, Carla Danese, Jay Chhablani, Paolo Lanzetta

**Affiliations:** 1 Department of Medicine-Ophthalmology, University of Udine, 33100 Udine, Italy; daniele.veritti@uniud.it (D.V.); valentina.sarao@uniud.it (V.S.); soppelsa.valentina@spes.uniud.it (V.S.); carla.danese@gmail.com (C.D.); 2 Istituto Europeo di Microchirurgia Oculare (IEMO), 33100 Udine, Italy; 3 Medical Retina and Vitreoretinal Surgery, University of Pittsburgh School of Medicine, Pittsburg, PA 15261, USA; jay.chhablani@gmail.com

**Keywords:** aflibercept, age-related macular degeneration, anti-VEGF, bevacizumab, brolucizumab, meta-analysis, meta-regression, ranibizumab

## Abstract

The use of anti-vascular endothelial growth factor (VEGF) agents has profoundly changed the prognosis of neovascular age-related macular degeneration (nAMD). As clinical experiences have accumulated, it has become mandatory to summarize data to give information that can be useful in everyday practice. We conducted a systematic review to identify randomized controlled trials (RCTs) and observational studies that reported 12-month changes in best-corrected visual acuity (BCVA) in patients with nAMD on anti-VEGF monotherapy. Data were analyzed in a random-effects meta-analysis with BCVA change as the primary outcome. Meta-regression was conducted to evaluate the impact of multiple covariates. Four hundred and twelve heterogeneous study populations (109,666 eyes) were included. Anti-VEGFs induced an overall improvement of +5.37 ETDRS letters at 12 months. Meta-regression showed that mean BCVA change was statistically greater for RCTs (*p* = 0.0032) in comparison with observational studies. Populations following a proactive regimen had better outcomes than those following a reactive treatment regimen. Mean BCVA change was greater in younger populations, with lower baseline BCVA and treated with a higher number of injections (*p* < 0.001). Our results confirm that anti-VEGFs may produce a significant functional improvement at 12 months in patients with nAMD.

## 1. Introduction

Neovascular age-related macular degeneration (nAMD) is the leading cause of irreversible vision loss among the over-50s living in developed countries, with a prevalence rate between 5.8% and 15.1% of the population, which constantly increases with age [1].

In recent times, intravitreal anti-vascular endothelial growth factor (anti-VEGF) therapy has become the treatment of choice for nAMD, supported by evidence from randomized clinical trials (RCTs) as well as routine clinical practice, demonstrating efficacy in preventing visual loss and improving vision [1,2]. Currently, three anti-VEGF drugs (ranibizumab, aflibercept, and brolucizumab) are authorized for the treatment of nAMD, whilst bevacizumab, developed and approved for different types of tumors, is widely employed in an off-label fashion in many countries. The magnitude of effect of anti-VEGF drugs on visual acuity was evident from the early monthly dosing trials. Later, studies based on a pro re nata (PRN) or a treat and extend (TAE) dosing strategy led to results that in some cases emulated those obtained with monthly dosing [2]. However, the published outcomes of real-world experiences show large variability, making it challenging to incorporate this evidence into clinical decision making. Treatment outcomes in routine practice may be different from what is obtained in RCTs. This can reflect the fact that study populations in RCTs are highly selective and may not entirely represent real-world patients. Moreover, patients in real-world clinical settings may be treated with dosing and/or regimens that differ from what recommended in the product’s label, mainly due to logistic problems and economic considerations [3,4,5,6]. Consequently, it is not clear to what extent the outcomes from RCTs can be replicated in everyday clinical practice. The objective of this study was to synthesize the evidence available about the efficacy of intravitreal anti-VEGFs for the treatment of nAMD based on a systematic review and a meta-analysis of published RCTs and observational/real-life studies. Moreover, we intended to identify clinical and study factors that may have an impact on the reporting of outcomes through a meta-regression model. Specifically, the aim of this work is to give an answer to the following ten questions:

Are results between RCTs and real-life/observational studies different?

1. Are results between RCTs and real-life/observational studies different, when analyzing each anti-VEGF agent?
2. Is the outcome influenced by the treatment regimen?
3. Is the outcome influenced by the treatment regimen, when considering only real-life/observational studies?
4. If proactive regimens produce better results, is this accurate when considering each anti-VEGF agent?
5. Is the outcome influenced by the frequency of treatments?
6. If the number of treatments has an effect on the results, is this accurate when considering each anti-VEGF agent?
7. Comprehensively, which agent shows more favorable results?
8. In real life/observational studies, which agent produces better results?
9. Are real-life visual results influenced by baseline characteristics?

## 2. Materials and Methods

A stepwise procedure, which includes a systematic literature review (SLR), a meta-analysis, and a meta-regression, was utilized to assess the efficacy/effectiveness of intravitreal therapy in patients affected by nAMD.

### 2.1. Systematic Literature Review

A SLR of available studies, which include patients affected by naïve nAMD and treated with intravitreal ranibizumab, aflibercept, bevacizumab, or brolucizumab with 52-week follow-up, was conducted. The present review was completed according to the protocols reported in the Cochrane Handbook for Systematic Review of Interventions (v5.1.0). The outcomes are expressed as reported in the Preferred Reporting Items for Systematic Review and Meta-Analyses (PRISMA) [7]. In brief, EMBASE, PubMed, and Cochrane databases were searched for papers until March 2021 independently by 3 authors (VSa, VSo, and CD). The research strategy was focused on a mix of medical subject headings and the keywords: “age-related macular degeneration”, “choroidal neovascularization”, “anti-VEGF”, “AMD”, “CNV”, “aflibercept”, “bevacizumab”, “ranibizumab”, and “brolucizumab”.

The review was restricted to clinical studies available in peer-reviewed, English language publications, and those published until March 2021. Conference abstracts/papers, editorials, proposals, reviews, notes, letters to authors, news, and commentaries were not included in the review. The reference lists from selected articles were inspected for additional publications. The risk of bias was estimated both quantitatively and qualitatively with the Downs and Black checklist.

### 2.2. Meta-Analysis

A meta-analysis of the outcomes obtained from the SLR was performed. Inclusion criteria for the meta-analysis consisted of studies including naïve nAMD patients treated with ranibizumab, aflibercept, bevacizumab, or brolucizumab in monotherapy and reported 1-year (±4 weeks) effectiveness outcomes. The main aim of this meta-analysis was to extract a pooled estimate for effectiveness (best-corrected visual acuity (BCVA) change from baseline to week 52 in Early Treatment Diabetic Retinopathy Study (ETDRS) letters). Visual acuities expressed in LogMAR unit or decimal scale were converted to ETDRS letters before performing statistical analysis. Randomized controlled trials, real-life prospective, and retrospective clinical studies were considered. Papers that investigated specific populations affected by retinal angiomatous proliferation, polypoidal choroidal vasculopathy, or fibrovascular pigment epithelial detachment were excluded from the analysis. Studies in which a specific type of anti-VEGF could not be extracted from the results were also not considered. Publications from the same author/organization that included duplicated data were not included.

The treatment strategy was categorized into one of three groups. Populations treated on a fixed protocol such as monthly or bimonthly were codified as fixed. Those being injected under a PRN interval were categorized as PRN and in the same manner TAE approaches constituted the TAE group.

Fixed-effects and random-effects models were utilized to obtain estimates. Heterogeneity was determined with the I^2^ statistic. Egger’s linear regression was used to evaluate publication bias along with visualization of funnel plots.

### 2.3. Meta-Regression and Moderators Selection

We performed a meta-regression analysis. Pre-selected primary moderators were chosen on the basis of existing evidence. Moderators of interest were age at baseline, baseline BCVA, study type (RCT, real-life/observational study), drug, number of injections, and treatment schedule. The output variable considered was mean BCVA change in ETDRS letters at 52 weeks (±4 weeks).

### 2.4. Compliance with Ethics Guidelines

The present study is based on previously published articles and does not imply any new studies of human participants. This work did not necessitate ethical approval as it did not include human participants or animal subjects.

## 3. Results

### 3.1. Study Selection

**Figure 1:**
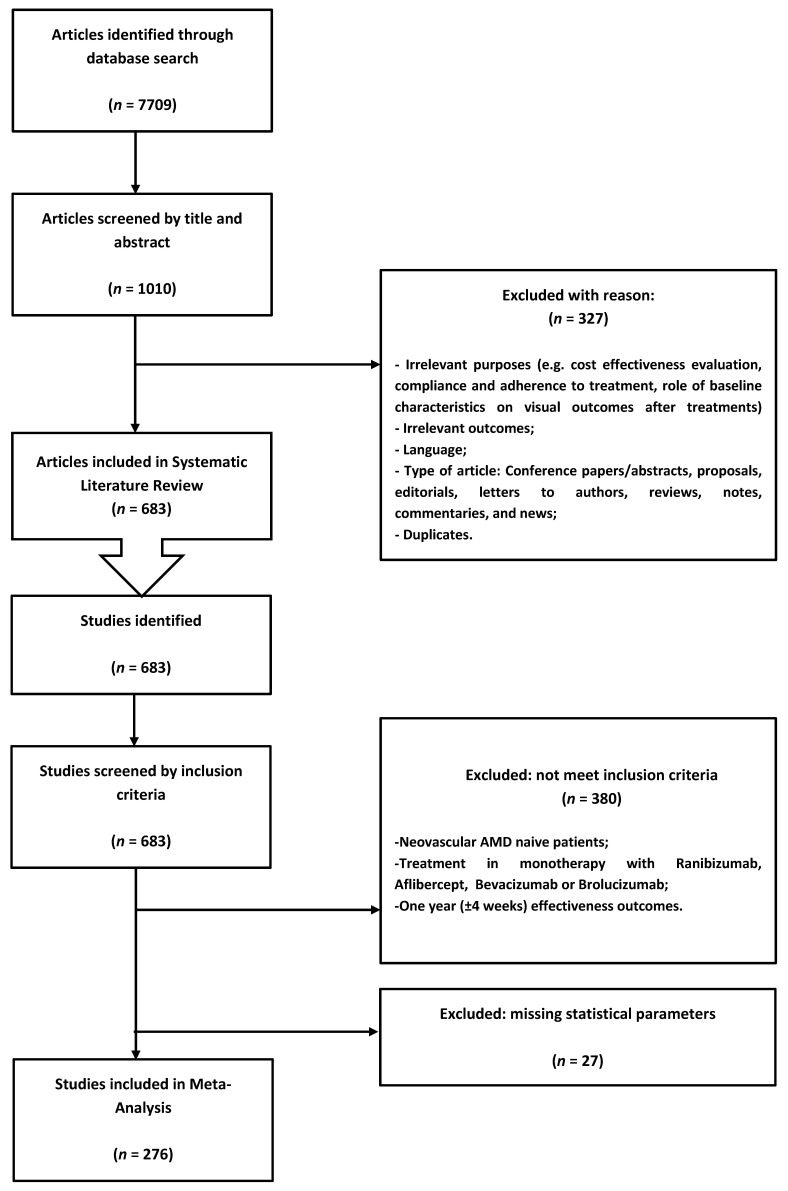
Flowchart of selection of studies and reason for exclusion.

The primary search produced 7709 reports. After screening of titles and abstracts and removal of duplicates, 683 potentially relevant papers were identified, and the full texts were extracted and individually screened for eligibility. Two hundred and seventy-six studies with 412 heterogeneous populations fulfilled inclusion criteria and were included in the analysis. [8,9,10,11,12,13,14,15,16,17,18,19,20,21,22,23,24,25,26,27,28,29,30,31,32,33,34,35,36,37,38,39,40,41,42,43,44,45,46,47,48,49,50,51,52,53,54,55,56,57,58,59,60,61,62,63,64,65,66,67,68,69,70,71,72,73,74,75,76,77,78,79,80,81,82,83,84,85,86,87,88,89,90,91,92,93,94,95,96,97,98,99,100,101,102,103,104,105,106,107,108,109,110,111,112,113,114,115,116,117,118,119,120,121,122,123,124,125,126,127,128,129,130,131,132,133,134,135,136,137,138,139,140,141,142,143,144,145,146,147,148,149,150,151,152,153,154,155,156,157,158,159,160,161,162,163,164,165,166,167,168,169,170,171,172,173,174,175,176,177,178,179,180,181,182,183,184,185,186,187,188,189,190,191,192,193,194,195,196,197,198,199,200,201,202,203,204,205,206,207,208,209,210,211,212,213,214,215,216,217,218,219,220,221,222,223,224,225,226,227,228,229,230,231,232,233,234,235,236,237,238,239,240,241,242,243,244,245,246,247,248,249,250,251,252,253,254,255,256,257,258,259,260,261,262,263,264,265,266,267,268,269,270,271,272,273,274,275,276,277,278,279,280,281,282,283]. The flowchart of selection steps is illustrated in Figure 1.

### 3.2. Study Characteristics

Attributes of the 276 analyzed studies are detailed in Table 1. Some studies consist of different heterogeneous study groups, which were considered as individual study populations in the present analysis. Most studies were real-life/observational, which was defined as single-arm interventional designs and retrospective chart reviews. Of the 276 studies included, 81 were randomized clinical trials, 95 were prospective cohort studies, and 100 were retrospective cohort studies. A total of 73 trials were conducted in Asia and Australia; 161 were conducted in Europe; and 42 in the United States. Less than half of the included studies (36%) were comparative. Four hundred and twelve heterogeneous study populations were found. The numerosity of the study populations ranged from 9 to 8598 eyes, with a total of 109,666 eyes enrolled. Specifically, 24,517 eyes were treated with aflibercept, 65,591 eyes with ranibizumab, 1038 eyes with brolucizumab and 18,520 eyes with bevacizumab. The relatively low number of subjects treated with brolucizumab is a consequence of the limited number of eligible studies available in literature so far. Therefore, this imbalance in sample size impacts the meta-regression analysis, limiting the sense of a comparison between brolucizumab and other anti-VEGFs. Nevertheless, brolucizumab data were included in the meta-analysis and in the meta-regression processes that did involve a comparison among drugs. Overall, the mean age varied from 63 to 90 years. Mean baseline BCVA ranged from 31 to 77 ETDRS letters. Two hundred and seventy-six studies were separately scored for their methodological quality using the Downs and Black checklist. Methodological quality ranged from 13 to 20, with a mean overall score of 17.3. For the most part, reduced quality across studies can be ascribed to poor reporting of blinding, loss to follow-up and characteristics of subjects lost to follow-up, randomization process, adjustment for confounding variables, and estimates of random variability.

### 3.3. Meta-Analysis

We found a high heterogeneity among studies considered in the analysis (I^2^ = 94.478%; *p* < 0.0001), and thereafter we chose a random-effects model. None of studies showed a significant effect on overall effect size, as showed by a leave-one-out sensitivity analysis. The meta-analysis provided an overall gain in BCVA of +5.37 ETDRS letters (95% CI: 5.01–5.72) at 12 months.

### 3.4. Meta-Regression

Several moderators showed robust effect modification. We applied a meta-regression process to provide answers to the following clinically significant questions.

1. Are results between RCTs and real-life/observational studies different?

A statistically significant (*p* = 0.0032) regression of difference in means on study type showed a coefficient of +1.32 ETDRS letters favoring RCTs over real-life/observational studies (CI 95%: +0.45; +2.20).

2. Are results between RCTs and real-life/observational studies different, when analyzing each anti-VEGF agent?

A statistically significant regression of difference in means on study type for aflibercept and ranibizumab (*p* = 0.042 and *p* = 0.0009, respectively) showed a coefficient of +1.80 (aflibercept) and +1.84 (ranibizumab) ETDRS letters advantaging RCTs over real-life/observational studies (CI 95%: +0.06; +3.53 for aflibercept and CI 95%: +0.75; +2.92 for ranibizumab). The same analysis performed for bevacizumab resulted in a not statistically significant difference (*p* = 0.95).

3. Is the outcome influenced by the treatment regimen?

Regression of difference in means on regimen was statistically significant, higher benefit was seen for fixed and TAE regimen over PRN regimen. Fixed regimen showed a coefficient of +2.23 ETDRS letters over PRN. (CI 95%: +1.32; +3.14; *p* < 0.0001). Treat and extend regimen showed a coefficient of +2.40 ETDRS letters over PRN. (CI 95%: +1.41; +3.39; *p* < 0.0001). No statistically significant difference was found between fixed and TAE regimen (*p* = 0.78).

4. Is the outcome influenced by the treatment regimen, when considering only real-life/observational studies?

Regression of difference in means on regimen was statistically significant, in favor of fixed and TAE regimen over PRN regimen, when including only real-life/observational studies. Fixed regimen showed a coefficient of +1.68 ETDRS letters over PRN. (CI 95%: +0.70; +2.67; *p* = 0.0008). Treat and extend regimen showed a coefficient of +2.02 ETDRS letters over PRN. (CI 95%: +0.98; +3.06; *p* = 0.0001). No statistically significant difference was found between fixed and TAE regimen (*p* = 0.61).

5. If proactive regimens produce better results, is this accurate when considering each anti-VEGF agent?

In patients treated with aflibercept, a statistically significant difference indicating more favorable results for fixed regimen over PRN regimen (coefficient +2.01 ETDRS letters; CI 95%: +0.62; +3.41; *p* = 0.005), and TAE regimen over PRN regimen (coefficient +2.58 ETDRS letters; CI 95%: +1.01; +4.15; *p* = 0.001) was described. Similarly, ranibizumab-treated populations had better outcomes in studies utilizing fixed regimen over PRN regimen (coefficient +2.47 ETDRS letters; CI 95%: +1.06; +3.88; *p* = 0.0006), and TAE regimen over PRN regimen (coefficient +2.33 ETDRS letters; CI 95%: +0.98; +3.69; *p* = 0.008). In patients treated with bevacizumab, regression of difference in means on regimen was not significant (*p* > 0.5).

6. Is the outcome influenced by the frequency of treatments?

A highly statistically significant effect resulted from regression of difference in means on mean number of treatments (coefficient +0.51 ETDRS letters; CI 95%: +0.34; +0.68; *p* < 0.0001).

7. If the number of treatments has an effect on the results, is this accurate when considering each anti-VEGF agent?

When looking at ranibizumab-treated populations, outcomes were significantly influenced by mean number of treatments (coefficient +0.69 ETDRS letters; CI 95%: +0.47; +0.91; *p* < 0.0001). The same analysis was not statistically significant for aflibercept (coefficient +0.32 ETDRS letters; CI 95%: −0.12; +0.76; *p* = 0.16) and bevacizumab (coefficient +0.16 ETDRS letters; CI 95%: −0.23; +0.55; *p* = 0.42).

8. Comprehensively, which agent shows more favorable results?

Regression of difference in means on drug showed that the studies employing aflibercept reported significantly superior results over ranibizumab (coefficient +1.78 ETDRS letters; CI 95%: +0.4; +4.15; *p* < 0.0001). A non-statistically significant trend for better results for aflibercept-treated populations over bevacizumab-treated populations was seen (coefficient +0.97 ETDRS letters; CI 95%: +0.09; +2.04; *p* = 0.07). The comparisons between the results published for brolucizumab-treated populations and the populations treated with other anti-VEGF agents were not statistically significant (*p* > 0.3). However, a non-significant trend towards better outcomes in the brolucizumab studies was detected (coefficient +0.03, +1.00, +1.78 ETDRS letters against aflibercept, bevacizumab, and ranibizumab, respectively).

When the same analyses were performed posing the treatment regimen as a precondition, no statistically significant differences among anti-VEGF drugs were found. In the populations treated with a fixed regimen, a trend to better outcomes (not statistically significant) was found for aflibercept (coefficient +0.87, +0.25 ETDRS letters over bevacizumab, and ranibizumab, respectively). When considering the populations treated with a PRN regimen, a trend to better outcomes (not statistically significant) was found for aflibercept (coefficient +0.04, +0.85 ETDRS letters over bevacizumab and ranibizumab, respectively). In the populations treated with a TAE regimen, a trend to better outcomes (not statistically significant) was found for aflibercept (coefficient +0.50, +0.89, and +1.01 ETDRS letters over bevacizumab, brolucizumab, and ranibizumab, respectively).

9. In real life/observational studies, which agent produces better results?

Aflibercept reported significantly better results over ranibizumab (coefficient +1.94 ETDRS letters; CI 95%: +1.05; +2.82; *p* < 0.0001), as shown by regression of difference in means on drug in real life/observational studies.

10. Are real-life visual results influenced by baseline characteristics?

Regression of difference in means was significant on age (coefficient −0.17 ETDRS letters; CI 95%: −0.26; −0.07; *p* < 0.001) and baseline BCVA (coefficient −0.11 ETDRS letters; CI 95%: −0.16; −0.07; *p* < 0.0001).

### 3.5. Publication Bias and Sensitivity Analysis

Funnel plot asymmetry was seen in the present meta-analysis. Egger’s linear regression (intercept = 3.11, *p* < 0.001) and by Begg’s rank correlation test (Kendall’s τ = 0.245, *p* < 0.001) also suggest the existence of publication bias. After imputing missing studies in the funnel plot, adjustment of effect size for possible publication bias using the trim-and-fill correction results in decreased, albeit still highly significant estimate of pooled mean difference (adjusted = +4.35 ETDRS letters; CI 95%: +4.02; +4.68; *p* < 0.0001). A ‘one-study-removed’ technique and a ‘cumulative meta-analysis’ technique were used to evaluate the potential influence of a small-study effect. Both techniques express negative results.

## 4. Discussion

Neovascular AMD is the main cause of vision loss in adult patients in developed countries [1,2]. The present study was conducted to synopsize the clinical evidence from RCTs and real-life/observational studies on functional results of intravitreal anti-VEGF treatment in the management of nAMD, obtaining a pooled estimate for BCVA change from baseline to week 52. This meta-analysis consists of 109,666 eyes and it is the largest and most comprehensive research to date that aim at synthetizing the clinical efficacy of intravitreal ranibizumab, aflibercept, bevacizumab, and brolucizumab in the treatment of nAMD at 12 months. The results obtained from this meta-analysis support the utilization of anti-VEGF agents as an effective therapeutic option for the treatment of nAMD, showing that significant BCVA gain is attainable. The present meta-analysis reports an overall increase in BCVA of approximately +5.3 ETDRS letters after one year of intravitreal anti-VEGF therapy. A high variability was found between studies, as demonstrated by the wide variance in pooled effect size (p heterogeneity, <0.0001). The interpretation of average effect size is increasingly complex as the presence of intertwined modifiers, independent predictors, and confounding variables multiplies. It remains an important goal to identify under what conditions anti-VEGF therapies may unlock their full potential. To elaborate on this matter, a meta-regression was carried out. RCTs showed an overall gain in visual acuity of +6.42 letters (95% CI: 5.50–7.33). Real-life/observational studies were calculated to have an increase of +5.01 letters (95% CI: 4.65–5.38). A statistically significant difference in BCVA was noticed between RCTs and real-life studies (*p* < 0.01) and, as expected, we found a higher variability in real-life results. This is in line with previous reports indicating that outcomes achieved with anti-VEGFs in real-life studies for the treatment of nAMD are not as good as those obtained in RCTs. However, it remains a matter of discussion whether a difference of +1.3 ETDRS letters is clinically meaningful. In the present meta-analysis and meta-regression, we choose a random-effects approach as the observed heterogeneity in the estimates may be attributed to between-study heterogeneity in true effects and within-study sampling error.

Growing evidence suggests that the regimen employed, and the frequency of anti-VEGF injections, have an impact on the visual outcome when treating a patient affected by nAMD [2]. Data from our analysis confirm this hypothesis. In detail, we found a statistically significant correlation between the number of anti-VEGF administrations and BCVA change (*p* < 0.0001). At month 12, each additional treatment induces a +0.51-letter gain. Yet, these results are not uniform among all anti-VEGF agents. The drug most dependent on the number of injections per year seems to be ranibizumab (coefficient +0.69 ETDRS letters per injection). We believe that this finding can be ascribed to both pharmacological properties and to the characteristics of the studies analyzed. In detail, the variability in the number of injections is much wider in ranibizumab studies than in those using aflibercept and bevacizumab. This is mainly because the larger part of ranibizumab studies apply a PRN regimen that involves a wider variability in the number of injections. Moreover, we investigated the role of the treatment regimen employed in obtaining the most favorable results. Results from the present meta-regression indicate that better outcomes are seen when employing a proactive treatment regimen (fixed or TAE) over a reactive treatment regimen (PRN). These results are also confirmed when analyzing real-life studies alone. Actually, many factors may interfere with the therapeutic efficacy of PRN treatment regimen in a real-life scenario, including administrative and logistic considerations. For example, improper appointment scheduling for treatment and monitoring visits is indeed a real-world factor that may result in unsatisfactory outcomes. Moreover, strict adherence to rigorous retreatment criteria is often difficult to obtain in a real-life scenario, due to inhomogeneity in imaging technologies and physicians’ knowledge and skills. This represents a limitation in maximizing visual gains, leading to suboptimal outcomes for the patients.

When analyzing baseline characteristics that may influence visual outcomes, our meta-regression showed that the 12-month BCVA change negatively correlated with baseline BCVA, which is consistent with prior experiences, revealing an inverse correlation between baseline BCVA and long-term BCVA change. Our analysis also revealed a negative correlation in BCVA change with increasing age. This negative correlation may be a consequence of worsened functional results at later age of presentation, when both the advanced stage of the disease and a decreased response to therapy may lead to inferior clinical outcomes. Key results from our work are reported in Table 2.

The main strength of the present work is that it provides an exhaustive and paradigmatic overview of the various therapeutic approaches used in real-life clinical practice and in RCTs for nAMD patients. We employed a predefined search strategy, three independent reviewers performed data extraction, and subgroup and sensitivity analysis were also conducted.

However, some limitations of the current study should not be ignored. First, the enrolled studies were limited to English language. This may have led to studies not being included, resulting in a not quite comprehensive data set. Second, the quality of included studies is variable. Real-life/observational studies exhibit a higher level of bias than RCT, including publication bias. Third, the heterogeneity among studies was notable, possibly due to confounding variables such as sample sizes, ethnic distribution of the study population, study designs, CNV types, and treatment modalities. Actually, uncontrolled confounding predisposes to bias when comparing observational studies and RCT. Fourth, the data used to establish these results might suffer from sample selection bias.

Finally, our results, from a methodological point of view, are also susceptible to ecological bias and study-level confounding, which means that the observed across-study relationships may not properly mirror the individual-level relationships within trials. In this sense, a network meta-analysis is probably less prone to misinterpretation. For all these motives, care must be exercised in conjecturing any form of quantitative relationship, which may alter over time and with a larger number of reports/studies included in the analysis.

## 5. Conclusions

In conclusion, the evidence for intravitreal therapy with anti-VEGF agents has been confirmed in this meta-analysis to be highly beneficial in the therapy of nAMD both in clinical trials and in real-life experiences. Frequency of injections and proactive treatment regimens are both factors related to best outcomes with currently available anti-VEGF agents.

## Figures and Tables

**Table 1:** Study Characteristics.

Study Type	Randomized Controlled Studies	Observational/ Real-Life Studies	Prospective Studies	Retrospective Studies
Eyes (populations)	27,785 (81)	81,881 (331)	39,008 (202)	70,288 (210)
Drug	Aflibercept	Ranibizumab	Brolucizumab	**Bevacizumab**
Eyes (populations)	24,517 (102)	65,591 (230)	1038 (3)	18,520 (77)
Regimen	Fixed	Pro-re-nata	Treat and Extend	
Eyes (populations)	13,318 (74)	81,651 (270)	7285 (57)	

**Table 2:** Efficacy of intravitreal anti-VEGFs for the treatment of neovascular AMD at 12 months: key results.

The use of anti-VEGF agents leads to a significant visual improvement in neovascular AMD patients.
Randomized clinical trials typically produce higher visual gains over real-life studies.
Proactive treatment regimen (fixed or treat-and-extend) usually leads to better outcomes over a reactive treatment regimen (pro-re-nata)
Frequency of anti-VEGF injections is a relevant factor and influences the visual outcome.
High baseline visual acuity and increased age reduce the functional response to intravitreal anti-VEGF therapy.

Legend: AMD: Age-related macular degeneration; VEGF: Vascular endothelial growth factor.

## Data Availability

Not applicable.
